# Cohort profile: Outcome Monitoring After Cardiac Surgery (OMACS) – a prospective UK cohort study of cardiac surgery patients at the Bristol Heart Institute

**DOI:** 10.1136/bmjopen-2024-091518

**Published:** 2025-02-05

**Authors:** Katherine Joyce, Rachel Maishman, Terrie Walker-Smith, Helena Smartt, Emma Hopkins, Penny Lambert, Gianni Angelini, Chris A Rogers, Barnaby Reeves, Rachel Brierley, Lucy Culliford

**Affiliations:** 1 Bristol Trials Centre, University of Bristol, Bristol, UK; 2 Bristol Heart Institute, Bristol, UK; 3 University of Bristol, Bristol, UK

**Keywords:** Cardiac surgery, Cardiothoracic surgery, Observational Study

## Abstract

**Purpose:**

The Outcome Monitoring After Cardiac Surgery (OMACS) cohort study was set-up with the aim of establishing a rich source of biological samples and health status data from patients who undergo cardiac surgery. The objectives were to use these data to inform the design of new clinical studies and to provide samples and data to answer research questions.

**Participants:**

Recruitment began on 23 May 2016 and ended 31 May 2022. Adult patients undergoing cardiac surgery at University Hospitals Bristol and Weston NHS Foundation Trust were screened and approached for consent. Participants could optionally consent to provide biological samples (urine, blood and waste tissue) in addition to data. A total of 4068 patients consented to participate in the study with 2027 consenting to donate samples. Participants were sent quality-of-life follow-up questionnaires at 3 and 12 months after surgery. The clinical data were collected from hospital records/databases.

**Findings to date:**

The OMACS population appears to be representative of the wider cardiac surgery population with similar preoperative demography to those reported on the UK surgery population. To date, eight studies have been carried out by research teams using data from a total of 1165 OMACS participants. Two Studies Within A Trial have been performed by the OMACS study team. The format of the study patient information leaflet was not significantly associated with an increased recruitment rate, and an alternative theory-informed cover letter included with the 12-month follow-up questionnaires was not associated with an increase in questionnaire completion. Additional exploratory research carried out within the OMACS study has been presented at international conferences.

**Future plans:**

The OMACS study is now closed. The cohort’s data and samples will be available to share with researchers, providing an opportunity to answer a variety of research questions (eg, evaluating predictors of adverse outcomes after cardiac surgery such as biomarkers, surgical methods and pre-existing conditions). The data and samples will be available for sharing in a linked anonymised format.

**Trial registration number:**

ISRCTN90204321 (date assigned: 21 January 2015).

**STRENGTHS AND LIMITATIONS OF THIS STUDY**

- The Outcome Monitoring After Cardiac Surgery (OMACS) study combined routinely collected data with prospectively collected data for items not well described in routine data sets, such as postoperative complications.
- The resultant data set and associated biobanked samples provide a rich source of information for future research into cardiac surgery outcomes.
- OMACS provided a host study for methodological research in the form of Studies Within A Trial.
- The study was based in a single cardiac surgery centre.
- Questionnaire follow-up rates were lower than expected, resulting in missing quality-of-life data to complement clinical data

## Introduction

 Cardiac surgery patients are often not closely monitored after discharge back to their general practitioner and surgical follow-up is fairly time-limited. In addition, clinical trials of surgical patients only collect the data needed to answer their specific research questions and follow-up is again limited to the scope of their trials. This means that there are few opportunities to characterise cardiac surgery patients in terms of their demographics, postoperative complications, outcomes and quality of life (QoL) outside of the scope of a specific clinical trial.

The Outcome Monitoring After Cardiac Surgery (OMACS) study aimed to collect short-, medium- and long-term health data and samples from patients undergoing cardiac surgery. The aim was to collect trial quality data to the point of discharge and QoL data up to 12 months and link these data to routine hospital data sets and long-term Hospital Episode Statistics (HES) data. Samples were also collected and banked for future analysis as part of a secondary research ethics committee approved study. These data and samples were intended to help characterise cardiac surgery patients, answer a broad range of secondary research questions and inform the design of future clinical trials. Data and samples were to be used in the following ways:

- To investigate predictors of early complications and late failure of interventions.
- To inform evaluations of the long-term effectiveness of interventions.
- To be made available to researchers to answer additional research questions (eg, the development of predictive indices for complications of cardiac surgery based on patient characteristics, clinical signs, epigenetic and metabolomic data).

The protocol has been published^1^ and this cohort profile describes how the patients were enrolled into the cohort study, their characteristics at enrolment and their outcomes, with the aim of publicising the existence of the resource and encouraging other researchers to apply to access the data to address specified research questions.

## Cohort description

### Study setting and population

The OMACS study aimed to recruit all patients who underwent cardiac surgery at the Bristol Heart Institute, University Hospitals Bristol and Weston NHS Foundation Trust, (BHI, UHBW). Adult patients (age 18 or older) undergoing surgery were invited to participate in the OMACS study between May 2016 and May 2022. Patients unable to give consent through mental incapacity, prisoners and patients whose main residence was outside the UK were excluded.

### Study timeline and consent options

Patients were approached for consent by an appropriately trained research nurse who was part of the direct care team. Two consent models were used during the OMACS study:

- Consent model 1—participants were contacted postoperatively by post and invited to consent to complete QoL questionnaires and for linkage to routinely collected hospital data. Participants had the option to return paper consent forms and receive questionnaires by post or to consent and complete questionnaires online.
- Consent model 2—participants were approached and consented to passive data collection about their hospital stay (including basic operative details, complications and ward movements) and linkage to routine data. Participants could also optionally consent to blood, tissue and urine samples being taken (if approached preoperatively) and to completing questionnaires. Patients were generally approached for consent preoperatively but could also be approached postoperatively if missed.

Consent model 1 was used between May 2016 and September 2017. Consent model 2 was used between June 2017 and May 2022.

**Figure 1:**
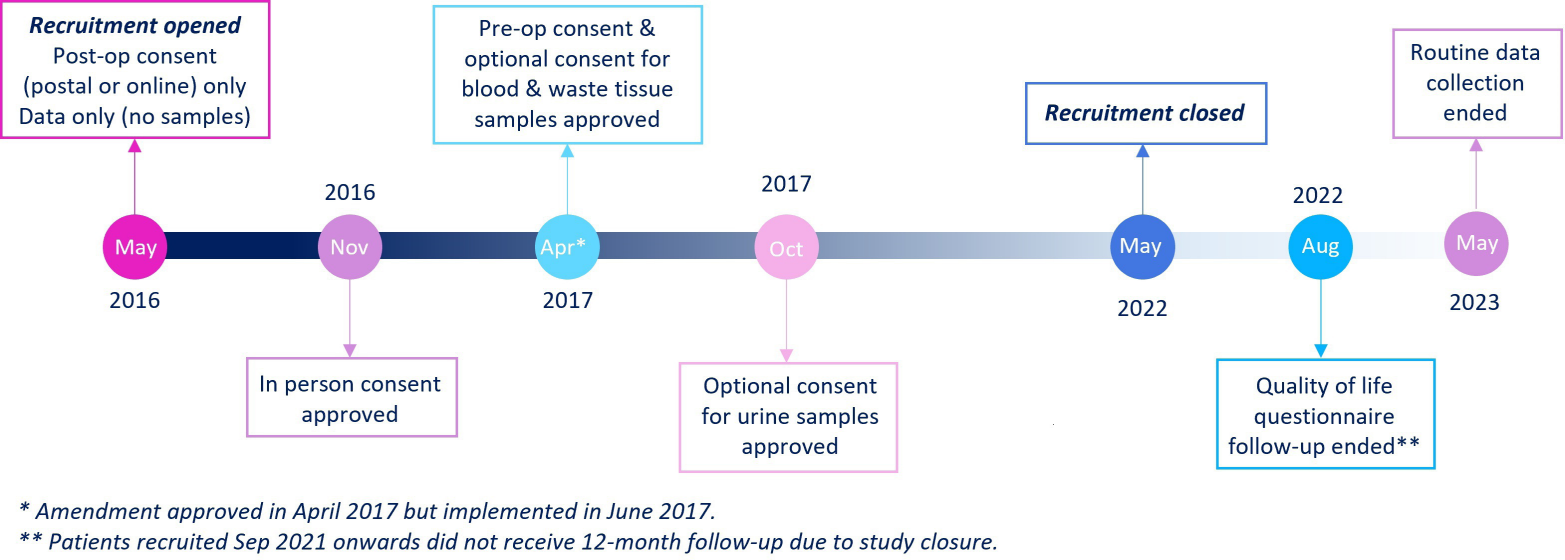
Outcome Monitoring After Cardiac Surgery study timeline.

Over the course of the study, several study amendments were approved to make the consent pathway more flexible and to include a collection of biological samples (see figure 1).

### Data collection and storage

The primary data source for the OMACS study was intraoperative details and postoperative outcome data entered directly onto the OMACS database. Data were extracted manually from patient records by study research nurses after participant discharge. QoL questionnaires were completed by participants at 3 and 12 months postoperatively. Participants who underwent coronary artery bypass grafting (CABG) surgery were asked to complete the Coronary Revascularisation Outcomes Questionnaire (CROQ), all other participants were asked to complete the Short form-12 (SF-12) questionnaire. These were completed on paper and returned to the clinical team for entry onto the OMACS database, with participants consented under consent model 1 given the option to complete questionnaires online.

Routine hospital data were also provided by the relevant UHBW teams to supplement the data collected on the OMACS database and minimise the number of data items for direct data entry. These included:

- Data on mortality after hospital discharge, provided by the critical care clinical information system (CIS) team.
- Preoperative and operative data, extracted from the patient administration and tracing system (PATS) (provided by the UHBW cardiac audit team) and the patient history database (PHD) (accessed by the Bristol Trials Centre (BTC) database team).
- Data routinely collected during the patient stay in the cardiac intensive care unit (CICU), provided by the critical care CIS team (the list of data items to be extracted was verified with the chief investigator and clinical colleagues and included data that were likely to be useful for further research but not collected within the OMACS database).

HES data could not be obtained for OMACS participants, due to the requirement of a specific research question when applying for HES data.

### Sample collection and storage

If participants consented to donate biological samples to the study, the samples listed in table 1 were collected (when logistics and staff capacity allowed, please note time points were flexible to maximise completeness of samples and the time of each sample was noted).

**Table 1:** Sample collection schedule

Sample type	Time point			Number of frozen aliquots per time point	Volume of aliquots	Number of frozen aliquots per patient
	Preoperative	2-hour postoperative	24-hour postoperative			
Plasma (EDTA)	Yes	Yes	Yes	6	500 µL	18
Buffy coat (taken from EDTA tube)	Yes	Yes	Yes	2	1 mL	6
Serum (Z Serum Clot Activator)	Yes	Yes	Yes	6	500 µL	18
PAXgene	Yes	Yes	Yes	1	2.5 mL	3
Urine	Yes	No	Yes	14	1 mL	28

EDTAEthylenediaminetetraacetic acid

Blood and urine samples were processed in UHBW laboratories before being frozen for storage. They were stored in the form of serum, plasma, buffy coat and whole blood in a PAXgene RNA tube (BD Biosciences). They were processed according to the below procedures for each time point/sample.

- Plasma and buffy coat: blood was collected in a 9 mL EDTA vacutainer. Blood was mixed with the anticoagulant by inverting the tube three times as soon as the sample was taken and placed on ice. The plasma fraction was prepared as soon as it was received in the laboratory, by centrifugation at 1500 g for 10 min at 4–5°C. The appearance of the sample was recorded as clear (CL), turbid (TU), haemolysed (HA) or haemolysed turbid (HT). If the layers were disturbed the sample would be centrifuged again at 1500 g for 2 min before pipetting off the plasma into up to 6500 µL aliquots, and the buffy coat layer into two aliquots. The buffy coat aliquots were topped up with red cells until each tube contained 1 mL. Aliquots were transferred to a −80°C freezer for long-term storage.
- Serum: blood was collected in a 9 mL Z Serum Clot Activator vacutainer. The vacutainer was left upright in a rack for 60 min to allow the blood to clot on ice. This was then centrifuged at 2500 g for 15 min at 4ºC. The appearance of the serum was recorded as CL, TU, HA or HT. The serum was transferred into up to 6500 µL aliquots (if less than 3 mL was obtained, fewer aliquots were filled). The aliquots were transferred to a −80ºC freezer for long-term storage.
- Whole blood with RNA preserved (PAXgene: blood was collected in a PAXgene RNA vacutainer (not put on ice) and transferred to the laboratory as soon as possible. The tube was kept upright at room temperature for a minimum of 2 hours (max 72 hours), transferred to a −20ºC freezer for 24 hours to 5 days, then transferred to a −80ºC freezer for long-term storage.
- Urine: collected in a 100 mL urine sample pot, placed on ice and transferred to the laboratory as soon as possible. The urine was spun within 6 hours of sampling, for 10 min at a minimum of 4500 g. The urine was then pipetted into 14 1 mL aliquots, then transferred to a −80°C freezer.

All samples were transferred in batches to a Health Technology Assessment licensed biobank (Bristol Bioresource Laboratories, University of Bristol) for long-term storage at −80°C.

Due to storage constraints, waste tissue was collected prospectively only when required for a specific ethically approved study. Therefore, no waste tissue is stored for future use.

### Recruitment progress

**Figure 2:**
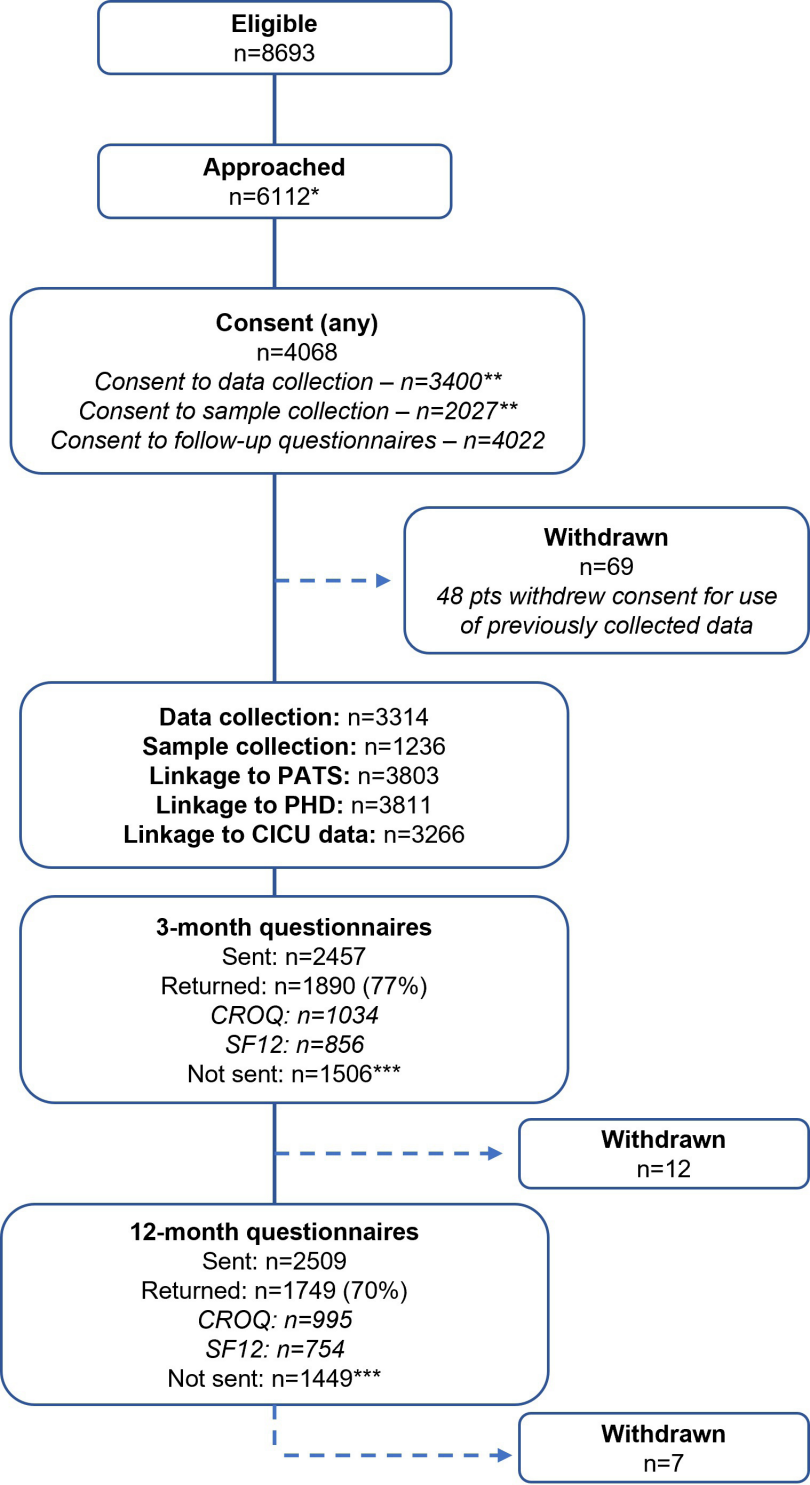
Study flow diagram. CICU, cardiac intensive care unit; CROQ, Coronary Revascularisation Outcomes Questionnaire; PATS, patient administration and tracing system; PHD, patient history database; SF-12, Short form-12.

The number of participants recruited into the OMACS study has been summarised in figure 2. When using consent model 1, all patients identified as eligible for OMACS were approached. When using consent model 2, some eligible patients were not approached due to staffing and logistical constraints.

Under consent model 1, 1517 patients were approached and 668/1517 (44%) patients consented to participate in OMACS. 97% (649/668) returned 3-month questionnaires with their consent forms. Questionnaires were not sent between November 2017 and April 2019 due to issues with the database. Of those participants that were sent 12-month questionnaires to complete, the questionnaires were returned by 268/310 (86%) of participants.

Under consent model 2, 4595/7176 (64%) eligible patients were approached. Of those approached, 3400/4595 (74%) consented to OMACS data collection, of whom 2027 (60%) consented to donate samples and 3354 (99%) consented to complete postoperative questionnaires. 3-month questionnaires were returned by 1241/1791 (69%) participants who were sent questionnaires, compared with 1481/2199 (67%) participants at 12 months.

Routine mortality data were obtained for 4008/4068 (99%) patients. PATS and PHD data were obtained for 3803/4068 (93%) and 3811/4068 (94%) of participants, respectively. CICU data was only requested for participants who consented to in-hospital data collection under consent model 2 and was obtained for 3266/3314 (99%) of those participants.

Recruitment and data and samples collection ended when the funding came to an end.

### Patient and public involvement

The Cardiovascular patient advisory group, funded by the Bristol Cardiovascular Biomedical Research Centre, was regularly consulted with about OMACS and also received regular updates.

### Participant characteristics

#### Demographics

The OMACS cohort was predominantly men (2463/3313, 74%) with a median age of 64.7 years and a mean body mass index of 28.2. Isolated CABG surgery was performed on 1227/3128 (39%) participants, with a further 946/3128 (30%) having isolated valve surgery, 308/3128 (10%) having combined CABG and valve surgery and 647/3128 (21%) having other cardiac procedures. The majority of participants had elective or urgent surgery (3090/3128, 98%). Previous myocardial infarction (MI) was reported in 697/2769 (25%) participants and 210/2905 (7%) participants had undergone previous cardiac surgery. 20% of the cohort were diabetic (573/2819) and 1927/2791 (69%) had a history of hypertension. The majority of the cohort had preoperative sinus rhythm (2399/2830, 85%) and good ejection fraction (left ventricular ejection fraction >50%) (2363/3045, 78%). Information on whether the participant has congenital heart disease (CHD) was added to the prospectively collected data set in 2020, and CHD was identified in 256 OMACS participants.

Demographic data are described both overall and separately for the subgroup of participants with samples taken. The subgroup of participants who provided samples are representative of the wider OMACS cohort (table 2).

**Table 2:** Demographic data summarised for the overall Outcome Monitoring After Cardiac Surgery cohort and separately for the subset of participants with samples collected

Demography	Overall(n=3314)		Participants with samples(n=1236)	
Age mean, (SD)	64.7	(12.99)	64.5	(12.84)
Male sex	2463/3313	(74)	914/1236	(74)
Surgical procedure				
Isolated CABG	1227/3128	(39)	440/1149	(38)
Isolated valve	946/3128	(30)	334/1149	(29)
Combined CABG and valve	308/3128	(10)	111/1149	(10)
Other cardiac procedures	647/3128	(21)	264/1149	(23)
Operative urgency				
Elective	1862/3139	(59)	687/1159	(59)
Urgent	1228/3139	(39)	471/1159	(41)
Emergency/salvage	49/3139	(2)	1/1159	(0.1)
BMI mean (SD)	28.2	(5.13)	28.3	(5.07)
Angina CCS class				
No angina	1097/2832	(39)	403/1060	(38)
I	316/2832	(11)	148/1060	(14)
II	800/2832	(28)	292/1060	(28)
III	430/2832	(15)	157/1060	(15)
IV	189/2832	(7)	60/1060	(6)
Dyspnoea status				
I	460/2829	(16)	175/1058	(17)
II	1337/2829	(47)	493/1058	(47)
III	933/2829	(33)	352/1058	(33)
IV	99/2829	(3)	38/1058	(4)
Previous MI	697/2769	(25)	256/1009	(25)
Previous PCI	325/2761	(12)	137/1003	(14)
Previous cardiac surgery	210/2905	(7)	82/1083	(8)
Diabetic	573/2819	(20)	231/1054	(22)
History of hypertension	1927/2791	(69)	749/1032	(73)
Smoking status				
Never smoked	1426/2795	(51)	528/1033	(51)
Ex-smoker	1155/2795	(41)	437/1033	(42)
Current smoker	214/2795	(8)	68/1033	(7)
Preoperative renal failure	24/2771	(1)	14/1029	(1)
History of pulmonary disease	303/2805	(11)	106/1047	(10)
History of neurological disease	232/2802	(8)	82/1044	(8)
History of neurological dysfunction	73/2809	(3)	26/1042	(2)
Extracardiac arteriopathy	285/2771	(10)	92/1008	(9)
Preoperative heart rhythm				
Sinus rhythm	2399/2830	(85)	875/1057	(83)
Atrial fibrillation/flutter	351/2830	(12)	147/1057	(14)
Complete heart block/pacing	48/2830	(2)	17/1057	(2)
Other abnormal rhythm	32/2830	(1)	18/1057	(2)
Left heart catheterisation	2614/3022	(86)	954/1113	(86)
Left main stem disease >50% diameter stenosis*	367/2737	(13)	119/1013	(12)
Ejection fraction				
Good (LVEF>50%)	2363/3045	(78)	860/1120	(77)
Fair (LVEF 31–50%)	580/3045	(19)	211/1120	(19)
Poor (LVEF≤30%)	102/3045	(3)	49/1120	(4)
Logistic EuroSCORE median (IQR)	3.1	(1.70–5.82)	2.9	(1.51–5.49)

Data are presented as n/N (%) unless otherwise specified.

Missing data (data given as numbers of participants with missing data overall (number of participants with samples with missing data)): age - 1 (0); BMI - 309 (104); logistic EuroSCORE - 710 (421).

* Other participants had either no left main stem disease or left main stem disease ≤50% diameter.

BMIbody mass indexCABGcoronary artery bypass graftCCSCanadian Cardiovascular SocietyLVEFleft ventricular ejection fractionMImyocardial infarctionPCIpercutaneous coronary intervention

Demographic data are summarised by operative procedure in online supplemental appendix table A1. Most noticeably, participants who underwent ‘other’ cardiac procedures were younger than those who underwent CABG and/or valve procedures (mean age 59.3 vs 63.9 or older for other operative procedures). In participants who underwent isolated valve surgery and other cardiac procedures, there were fewer men (64% in each vs ≥85% in the other operative procedures) and a higher proportion of participants who underwent elective surgery (78% and 70%, respectively, compared with 40% in the isolated CABG group and 58% in the combined CABG and valve group).

#### Operative details

The majority of participants in the cohort underwent cardiac surgery using cardiopulmonary bypass (2612/3047, 86%) with a median bypass time of 95 min (IQR 73–126) and median cross-clamp time of 65 min (47–91). Most operations were performed using cold blood cardioplegia with intermittent antegrade infusion. The majority of participants were given tranexamic acid (2533/2920, 87%). Red blood cell (RBC) transfusion was required for 332/2555 (13%) participants, fresh frozen plasma (FFP) for 170/2555 (7%), platelets for 506/2556 (20%) and cryoprecipitate for 214/2556 (8%). Arrhythmias at the end of the operation were reported for 833/3020 (28%) participants and pacing at the end of the operation was required for 888/3024 (29%). The median ventilation time was 11.5 hours (IQR 9.2–17.0), with reintubation required for 134/3269 (4%) participants. Of those undergoing CABG, most had two or three distal coronary anastomoses, with a small number 20/1482 (1%) reported as having no coronary anastomoses likely to be due to an error in coding the operative details. Full operative details are presented in table 3. Again, data are summarised separately for the participants with samples and this subset is a representative sample of the OMACS cohort.

**Table 3:** Operative data summarised for the overall Outcome Monitoring After Cardiac Surgery cohort and separately for the subset of participants with samples collected

Operative details	Overall(n=3314)		Participants with samples(n=1236)	
Number of distal coronary anastomoses (CABG patients only)				
0	20/1482	(1)	3/520	(1)
1	274/1482	(18)	107/520	(21)
2	521/1482	(35)	181/520	(35)
3	576/1482	(39)	193/520	(37)
4+	91/1482	(6)	36/520	(7)
Surgery performed using cardiopulmonary bypass	2612/3047	(86)	982/1125	(87)
Cumulative bypass time mean (SD)	95.0	(73.00–126.00)	100.5	(76.00–133.00)
Cumulative cross-clamp time median (IQR)	65.0	(47.00–91.00)	68.0	(49.00–94.00)
Cold cardioplegia*	2196/2578	(85)	836/970	(86)
Antegrade cardioplegia infusion†	2162/2567	(84)	821/964	(85)
Intermittent cardioplegia‡	2565/2573	(99.7)	962/967	(99.5)
Blood cardioplegia§	2575/2584	(99.7)	968/972	(99.6)
Tranexamic acid	2533/2920	(87)	953/1107	(86)
Cell saver set-up	762/2971	(26)	323/1119	(29)
RBC transfused intraoperatively	332/2555	(13)	139/979	(14)
FFP transfused intraoperatively	170/2555	(7)	71/979	(7)
Platelets transfused intraoperatively	506/2556	(20)	232/979	(24)
Cryoprecipitate transfused intraoperatively	214/2556	(8)	110/979	(11)
Pump blood returned	1110/2469	(45)	358/975	(37)
Activation factor VII	88/3005	(3)	39/1121	(3)
Arrhythmias at the end of the operation requiring treatment				
No, sinus rhythm only	2187/3020	(72)	802/1124	(71)
AV block	87/3020	(3)	36/1124	(3)
Atrial fibrillation/flutter	136/3020	(5)	46/1124	(4)
Ventricular fibrillation/ventricular tachycardia	25/3020	(0.8)	6/1124	(0.5)
Sinus bradycardia	220/3020	(7)	85/1124	(8)
Other	365/3020	(12)	149/1124	(13)
Pacing at the end of the operation				
None	2136/3024	(71)	792/1124	(70)
Single chamber	490/3024	(16)	202/1124	(18)
Dual chamber	313/3024	(10)	101/1124	(9)
Permanent	73/3024	(2)	25/1124	(2)
Other	12/3024	(0.4)	4/1124	(0.4)
Total ventilation time median (IQR)	11.5	(9.2–17.0)	11.8	(9.3–18.2)
Not extubated	18/3034	(1)	10/1122	(1)
Reintubation	134/3269	(4)	51/1221	(4)

Data are presented as n/N (%) unless otherwise specified.

Missing data (data given as numbers of participants with missing data overall (number of participants with samples with missing data)): cumulative bypass time - 21 (2); cumulative cross-clamp time - 39 (10); total ventilation time - 173 (50).

* All other participants had warm cardioplegia.

† All other participants had retrograde and antegrade CPB.

‡ All other participants had continuous CPB.

§ All other participants had crystalloid or other cardioplegia solution.

AVatrioventricularCABGcoronary artery bypass graftCPBcardiopulmonary bypassFFPfresh frozen plasmaRBCred blood cell

Operative details are split across the different operative procedures in online supplemental appendix table A2. Surgery was performed without cardiopulmonary bypass for approximately 35% of participants who underwent isolated CABG surgery. Cumulative bypass time and cross-clamp time were longest with other cardiac procedures (median 129 min and 89 min, respectively) and shortest in the isolated CABG participants (median 75 min and 45 min, respectively).

Surgery was performed using either warm or cold blood cardioplegia in the isolated CABG participants, compared with cold blood only for all other participants. Tranexamic acid was used for fewer participants undergoing other cardiac procedures (71%) compared with the other operative subgroups (>87% across the other subgroups). More participants had cell saver set-up in this subgroup also (41% compared with <30% for all other subgroups).

Blood product use, including RBC, FFP, platelets and cryoprecipitate, was lowest in the isolated CABG and isolated valve subgroups. Fewer participants had arrhythmias at the end of surgery in the isolated CABG subgroup (12% compared with 35% to 43% in the other subgroups). Similarly, fewer participants had pacing at the end of the operation in this subgroup (10%) compared with >38% in all other subgroups.

#### Postoperative outcomes

Patient outcomes have been described for the whole OMACS cohort, and for the subgroup of participants with samples taken (tables4  5). Major adverse cardiovascular events (MACE), defined as confirmed MI, stroke or death (any cause) before discharge, occurred in 3% of OMACS participants with a median of 2 days (IQR 1–5) from operation to first MACE event. Stroke was reported in 2% of OMACS participants and confirmed MI and in-hospital death in 1% of participants. 3% of participants died within 1 year of operation, with a median survival time of 63 days (IQR 9–183). Participants had a median ICU stay of 68.7 hours (IQR 46.3–111.3) and a median hospital stay of 7 days (IQR 5–10). Study retention was good, with 77% (1887/2454) of participants returning questionnaires at 3 months and 70% (1746/2506) at 12 months.

**Table 4:** Patient cardiovascular outcomes, mortality and length of stay summarised for the overall Outcome Monitoring After Cardiac Surgery cohort and separately for the subset of participants with samples collected

Patient outcomes	Overall(n=3314)		Participants with samples(n=1236)	
MACE	110/3272	(3)	56/1231	(5)
Time to MACE (days) median (IQR)	2.0	(1.0–5.0)	3.0	(1.0–6.5)
Confirmed MI	30/3273	(1)	14/1230	(1)
Stroke	52/3282	(2)	26/1230	(2)
In-hospital death	35/3305	(1)	22/1233	(2)
Time to in-hospital death (days) median (IQR)	6.0	(1.0–14.0)	7.5	(1.0–14.0)
Death within 1 year	99/3313	3	46/1236	4
Time to death (within 1 year) (days) median (IQR)	63.0	(9.00–183.00)	21.5	(6.00–108.00)
Time to ICU discharge (hours) median (IQR)	68.7	(46.30–111.30)	67.9	(44.90–111.20)
Time to hospital discharge (days) median (IQR)	7.0	(5.00–10.00)	7.0	(5.00–10.00)
Reoperation	168/3295	(5)	68/1231	(6)
Reoperation for bleeding	95/135	(70)	42/53	(79)
Reoperation for cardiac tamponade	24/135	(18)	6/53	(11)
Reoperation for cardiac arrest	7/136	(5)	5/54	(9)
Reoperation for low cardiac output	28/135	(21)	14/53	(26)
Reoperation, other reason	23/136	(17)	6/54	(11)
Cardiac arrest	38/3284	(1)	20/1230	(2)
Resuscitation attempted	34/38	(89)	18/20	(90)
Resuscitation successful	28/34	(82)	15/18	(83)
SVT/AF	1141/3284	(35)	426/1230	(35)
VT/VF	38/3283	(1)	21/1230	(2)
New pacing	904/3285	(28)	357/1230	(29)
Temporary pacing became permanent	119/859	(14)	46/341	(13)
ICD	26/2387	(1)	13/1120	(1)
Vasopressors used	1938/3034	(64)	770/1121	(69)
Any inotropes used	965/3030	(32)	370/1121	(33)
IABP inserted	36/3280	(1)	20/1230	(2)
Pulmonary artery catheter inserted	34/3280	(1)	17/1230	(1)
Vasodilator used	716/3281	(22)	287/1230	(23)
Tracheostomy	37/3281	(1)	18/1230	(1)
Mask CPAP	274/3131	(9)	86/1190	(7)
ARDS	13/3281	(0.4)	5/1230	(0.4)
Pneumothorax or pleural effusion requiring drainage	121/3031	(4)	53/1120	(5)
High flow oxygen	333/1967	(17)	169/870	(19)
TIA	10/3282	(0.3)	<5/1230	(0.2)
DVT	9/3281	(0.3)	<5/1230	(0.3)
Pulmonary embolus	10/3281	(0.3)	5/1230	(0.4)
Excess bleeding not requiring re-operation	50/3032	(2)	23/1121	(2)
Pericardial effusion requiring drainage	134/3131	(4)	66/1191	(6)

Data are presented as n/N (%) unless otherwise specified.

Missing data (data given as numbers of participants with missing data overall (number of participants with samples with missing data)): time to discharge - 1 (0); time to ICU discharge - 81 (18).

Counts of 4 or less have been suppressed to maintain anonymity.

ARDSacute respiratory distress syndromeCPAPcontinuous positive airway pressureDVTdeep vein thrombosisIABPintra-aortic balloon pumpICDimplantable cardioverter defibrillatorICUintensive care unitMACEmajor adverse cardiovascular eventMImyocardial infarctionSVT/AFsupraventricular tachycardia/atrial fibrillationTIAtransient ischaemic attackVT/VFventricular tachycardia/ventricular fibrillation

**Table 5:** Patient renal, GI and infection outcomes summarised for the overall Outcome Monitoring After Cardiac Surgery cohort and separately for the subset of participants with samples collected

Patient outcomes	Overall(n=3314)		Participants with samples(n=1236)	
Haemofiltration/dialysis since heart operation	73/3133	(2)	37/1191	(3)
Acute kidney injury	747/2967	(25)	302/1121	(27)
Stage 1	521/745	(70)	192/302	(64)
Stage 2	118/745	(16)	55/302	(18)
Stage 3	106/745	(14)	55/302	(18)
Peptic ulcer/GI bleed/perforation	13/3282	(0.4)	4/1230	(0.3)
Pancreatitis	<5/3282	(0.1)	<5/1230	(0.2)
Ischaemic bowel requiring treatment	<5/3282	(0.1)	<5/1230	(0.1)
Any unexpected complication	673/3308	(20)	300/1233	(24)
Infectious complications				
Any suspected infection	1050/2677	(39)	417/1029	(41)
Any confirmed infection	220/2310	(10)	104/904	(12)
Suspected sepsis	782/3030	(26)	310/1121	(28)
Temperature <36°C or >38°C	651/782	(83)	269/310	(87)
Unexplained increased heart rate above normal for the patient	186/780	(24)	79/310	(25)
CRP>5 mg/L	*773/782*	(99)	308/310	(99)
WBC>12.0	365/782	(47)	147/310	(47)
Unexplained increased respiratory rate above normal for patient	261/779	(34)	104/309	(34)
Respiratory infection	640/2662	(24)	256/1026	(25)
Superficial wound infection	128/2641	(5)	49/1022	(5)
Wound dehiscence requiring rewiring or treatment	29/2642	(1)	9/1022	(1)
UTI	43/2643	(2)	18/1023	(2)
Unspecified infection	48/2640	(2)	13/1022	(1)
Other infection	58/1878	(3)	26/790	(3)
Postoperative antibiotics started (participants with any suspected infection)	756/1048	(72)	303/416	(73)

Data are presented as n/N (%) unless otherwise specified.

Counts of 4 or less have been suppressed to maintain anonymity.

CRPC-reactive proteinGIgastrointestinalUTIurinary tract infectionWBCwhite blood cell

Postoperative complications reported in the whole OMACS cohort, and in the subset of participants with samples taken, are presented in tables4  5. Use of vasopressors (64%), inotropes (32%) and vasodilators (22%) were relatively common. The most commonly reported adverse events included supraventricular tachycardia/atrial fibrillation (35%) and new pacing (28%). High flow oxygen was required in 17% of participants. Acute kidney injury (AKI) was reported in 25% of participants, with stage 1 AKI most common (70%) and stages 2 and 3 AKI in approximately 15% of patients each. Suspected infections were commonly reported (39%) with suspected respiratory infections most common (24%). Postoperative antibiotics were given to 72% of participants with a suspected infection. Only 10% of participants with suspected infections had their infections confirmed by blood cultures. Complications that were collected as ‘free text’ data as they were not listed on the data collection forms as specific data items, were reported in 20% of participants. Details on these ‘other’ complications were collected and are available in the study data set, but have not been reported here. Complication rates were consistent between the whole cohort and the subset of participants with samples taken.

Summary scores were calculated for each of the postoperative QoL questionnaires and described in table 6

**Table 6:** Patient quality of life outcomes summarised for the overall Outcome Monitoring After Cardiac Surgery cohort and separately for the subset of participants with samples collected

Patient outcomes	Overall(n=3975)		Participants with samples(n=1225)	
SF-12 questionnaires at 3 months				
Questionnaires returned	856/1147	75	293/423	69
Physical component score median (IQR)	46.6	(39.37–53.66)	47.5	(39.37–53.87)
Mental component score median (IQR)	51.5	(42.69–57.71)	50.1	(42.35–57.56)
SF-12 questionnaires at 12 months				
Questionnaires returned	754/1169	64	243/368	66
Physical component score median (IQR)	49.7	(40.47–55.85)	49.5	(40.99–55.88)
Mental component score median (IQR)	53.2	(43.74–58.12)	52.4	(42.23–57.45)
Coronary Revascularisation Outcomes Questionnaire at 3 months				
Questionnaires returned	1031/1307	79	372/486	77
Symptoms score median (IQR)	92.9	(85.71–100.00)	92.9	(83.92–100.00)
Physical score median (IQR)	87.5	(71.43–100.00)	87.5	(68.75–100.00)
Cognitive function score median (IQR)	93.3	(80.00–100.00)	93.3	(73.33–100.00)
Psychological function score median (IQR)	83.9	(69.64–94.64)	83.9	(69.64–92.86)
Satisfaction score median (IQR)	83.3	(68.06–91.67)	80.3	(65.14–90.97)
Adverse events score median (IQR)	88.6	(77.27–95.45)	88.6	(79.55–95.45)
Coronary Revascularisation Outcomes Questionnaire at 12 months				
Questionnaires returned	992/1337	74	347/472	74
Symptoms score median (IQR)	96.4	(85.71–100.00)	96.4	(85.71–100.00)
Physical score median (IQR)	93.8	(75.00–100.00)	93.8	(75.00–100.00)
Cognitive function score median (IQR)	93.3	(80.00–100.00)	93.3	(80.00–100.00)
Psychological function score median (IQR)	91.1	(78.57–98.21)	91.1	(78.57–98.21)
Satisfaction score median (IQR)	83.3	(68.33–94.44)	83.3	(66.67–100.00)
Adverse events score median (IQR)	93.2	(86.36–97.73)	95.5	(86.36–97.73)

Data are presented as n/N (%) unless otherwise specified.

Missing data (data given as numbers of participants with missing data overall (number of participants with samples with missing data)): 3-month SF-12 summary scores - 7 (4); 12-month SF-12 summary scores - 6 (3); 3-month CROQ symptoms score - 4 (0); physical score - 14 22); cognitive function score - 7 (0); psychological function score - 8 (0); satisfaction score - 3 (0); adverse events score - 13 (1); 12-month CROQ symptoms score - 4 (1); physical score - 14 (3); cognitive function score - 6 (3); psychological function score - 8 (1); satisfaction score - 6 (1); adverse events score - 13 (3).

CROQCoronary Revascularisation Outcomes QuestionnaireIQRInterquartile rangeSF-12Short form-12

for the whole population and for the subgroup of participants with samples taken. As with the other outcomes, the subgroup of participants with samples appears to be representative of the wider OMACS cohort. Due to a database error, a small number of participants were sent the wrong questionnaire in error (45 CABG participants sent the SF-12 questionnaire and 68 non-CABG participants sent the CROQ).

Patient outcomes are compared across surgical procedures in online supplemental appendix table A3. MACE occurred in fewer isolated CABG participants (1% compared with 3%, 7% and 6% in the isolated valve, combined CABG and valve and other cardiac procedure subgroups, respectively). A longer time to MACE was observed in the combined CABG and valve participants (6 days, compared with 3 or fewer in the other operative subgroups). Participants who died before discharge in the combined CABG and valve subgroup had a longer time to death (median 10 days compared with 6 or less in the other subgroups). Participants who underwent combined CABG and valve surgery and other cardiac procedures had a longer ICU stay (median stay 91 hours and 89 hours compared with approximately 70 hours in the other subgroups), and longer overall hospital stay 8 days compared with 6 and 7 days for isolated CABG and isolated valve, respectively. Overall, complication rates were lower in the isolated CABG subgroup. Most noticeably, postoperative pacing was required in 10% of isolated CABG participants compared with >40% in the other operative subgroups.

No differences in questionnaire return rates across operative subgroups were observed (online supplemental appendix table A4). SF-12 summary scores were similar across the operative subgroups. CROQ physical scores at 12 months were higher in participants who underwent isolated CABG compared with those who underwent combined CABG and valve surgery or other cardiac procedures (median 100 compared with 93.8 in the other two subgroups). Conversely, cognitive function scores were highest in the combined CABG and valve subgroup (median 100 compared with 93.3 in both the isolated CABG subgroup and the subgroup of participants who underwent other cardiac procedures). Other summary scores were similar across the operative subgroups.

### Availability of samples

Samples were taken for 1236 participants. Baseline and 24-hour blood samples were taken for 1225 participants (99%). Six per cent of participants did not have 2-hour samples taken. Urine samples at baseline and 24 hours were not collected for 7% of participants; consent to urine samples was introduced later in the study after sample collection of EDTA and serum had already commenced.

## Findings to date

### Comparison to wider population

The OMACS population appears to be representative of the wider cardiac surgery population as reported in the Society for Cardiothoracic Surgey (SCTS) cardiac blue book 2020,^2^ which reported a mean age of 66 years, poor left ventricular function in 6% of patients and emergency/salvage procedures in <2.5% of patients amongst isolated CABG patients.^1^ A higher proportion of isolated CABG patients had a previous MI in the OMACS population (51%) compared with the wider isolated CABG population (18%). In-hospital mortality was similar between the isolated CABG patients and the wider cardiac surgery population (<1% in OMACS compared with 1% in the wider patient population) with an average hospital length of stay of 6 days in both.

The OMACS cohort appears to be representative of the wider UK cardiac surgery population as detailed in the 2002 National Institute for Cardiovascular Outcomes Research (NICOR) report.^3^ In the UK population the mean age of patients undergoing cardiac surgery ranges from 66.6 years in 2103/2014 to 65.1 years in 2020/2021, in OMACS the mean age was 64.7 years. The proportion of men was 74% in OMACS and in the UK population ranged from 73.5% in 2018/2019 to 75.0% in 2020/2021. This latter similarity is significant as research has established that women are under-represented in much cardiac surgery research.^4^

CROQ scores were compared against the scores calculated in the development of the CROQ instrument.^5^ CROQ summary scores in the OMACS cohort were slightly higher than those reported in the development cohort. Focusing on the 3-month follow-up scores, the OMACS cohort had a median symptom score of 92.9 compared with a mean score of 87.6 in the development cohort. The differences between scores were consistent across all summary scores. SF-12 summary scores were compared with those reported in a study of CABG patients operated on across 18 centres in Germany with coronary heart disease (https://www.ncbi.nlm.nih.gov/pmc/articles/PMC1768233/); while the SF-12 questionnaire was only administered to participants who did not undergo CABG surgery in OMACS, this was the most similar participant population available for comparison. SF-12 scores were similar between the OMACS population and the German population, with the greatest difference observed in the physical component score at 12 months (median 49.6 in OMACS compared with 43 in the CABG patients operated on in Germany). This difference might be due to the difference in operative procedures resulting in a different recovery process.

### Secondary research

The OMACS study provides an excellent resource for study teams to conduct important research on characteristics, outcomes, complications and other important questions relating to cardiac surgery patients. To date, eight studies have been carried out using data from a total of 1165 OMACS participants, of which samples have been provided for 532 participants; the results of these studies will be reported by the study teams carrying out the research. OMACS remains a source of both data and samples that can be shared to answer important clinical questions identified in the future. Appropriate research ethics committee approval must be in place for the use of the OMACS data and samples by secondary researchers. Any requests for samples or data should be directed towards the corresponding author.

### Study Within A Trial

Two Study Within A Trial (SWATs) have been performed and analysed within the OMACS study. First, an SWAT to investigate the impact of different styles of patient information leaflet (PIL) on recruitment rate.^6^ Recruitment rates were similar across the PIL styles; however, it was concluded that a more expensive booklet style PIL did not improve recruitment and therefore should not be implemented in future studies due to the increased costs associated with producing booklets.^5^ Second, an SWAT from the Northern Ireland Network for Trial Methodology Research SWAT repository (SWAT 24)^7^ was embedded into the study. This SWAT investigated whether study retention at 12 months (assessed by questionnaire return rates) was improved using a theory-informed cover letter compared with the usual letter. The theory-informed cover letter did not improve study retention at 12 months for the OMACS participants (results to be published).

### Conference contributions

In addition to published research, the OMACS data have been used to explore questions of interest, including whether data on operative procedures are recorded consistently across different routine data sources (PATS and PHD). For OMACs participants who had a CABG or aortic/mitral valve replacement operation in 2020, of the 461 participants who had operative records in both data sources, there were 433 (94%) records where there was agreement in type of operation and on manual checking of operation notes for the 28 discrepant cases, PHD was correct in 11/28 cases, PATs correct in 16/28 cases and there was one case where neither PATs or PHD were correct.^8^

### Strengths and limitations

The OMACS data provides a rich data source that could be interrogated when designing future cardiac surgery research.

The OMACS study has produced a bank of clinical data and samples for cardiac surgery patients which will allow a multitude of research questions to be answered by secondary researchers. These data and samples have already been used to investigate a number of research questions by secondary research teams, with a large volume of samples in storage available for further secondary research studies.

The scale of the OMACS study allowed inclusion of SWATs with minor amendments to the study.

The linkage to routine data sources such as CICU data is one of the strengths of the OMACS study. The successful linkage demonstrates that it is possible to obtain a large amount of additional data on patient recovery with to relieve the burden of prospectively collected data by research nurses and data collection staff. Unfortunately, the linkage to HES data could not be obtained for the OMACS cohort, however, this was due to a lack of specific research question. This means that long-term post-discharge outcomes except QoL are not available for this cohort. One limitation of the routine data is the potentially higher levels of missingness in the data; during direct data entry a great deal of effort is spent to minimise the level of missingness, however when using routine data sources these missing data are difficult to recover. While it has been demonstrated that routine data sources can be used in the place of direct data capture, this is not recommended for key study outcomes for this reason.

Further linkage of data is not possible, which limits any research to the data items collected and reported above.

## Collaboration

Those wishing to the data and samples for their research should contact the corresponding author who will provide a copy of the full data and sample access policy. In short, any reasonable request will be honoured unless prevented by sample depletion.

## supplementary material

10.1136/bmjopen-2024-091518 online supplemental file 1

## Data Availability

Data are available upon reasonable request.
