# Supplementary material for: Cohort profile: Outcome Monitoring After Cardiac Surgery (OMACS) – a prospective UK cohort study of cardiac surgery patients at the Bristol Heart Institute
Source: BMJ Open. 2025 Feb 5;15(2):e091518. doi: 10.1136/bmjopen-2024-091518 (PMC11800208; doi:10.1136/bmjopen-2024-091518)
Supplement: online supplemental file 1 [file bmjopen-15-2-s001.docx]

**Table A1:** Demographic data summarised for each of the operative subgroups in the OMACS cohort. Data are presented as n/N (%) unless otherwise specified.

| Demography | | Isolated CABG (n=1227) | | Isolated valve (n=946) | | Combined CABG and valve (n=308) | | Other cardiac procedures (n=647) | |
| --- | --- | --- | --- | --- | --- | --- | --- | --- | --- |
| Age *mean (SD)* | | 66.0 | (9.2) | 63.9 | (14.9) | 71.6 | (8.3) | 59.3 | (16.1) |
| Male | | 1043/1227 | (85%) | 602/945 | (64%) | 264/308 | (86%) | 417/647 | (64%) |
| Operative urgency | |  |  |  |  |  |  |  |  |
|  | Elective | 485/1226 | (40%) | 737/944 | (78% | 178/307 | (58%) | 449/646 | (70%) |
|  | Urgent | 723/1226 | (59%) | 200/944 | (21% | 125/307 | (41%) | 177/646 | (27%) |
|  | Emergency/salvage | 18/1226 | (1%) | 7/944 | (1%) | 4/307 | (1%) | 20/646 | (3%) |
| BMI *mean (SD)* | | 28.9 | (4.9) | 27.7 | (5.4) | 28.7 | (4.5) | 27.6 | (5.4) |
| Angina CCS class | |  |  |  |  |  |  |  |  |
|  | No angina | 124/1087 | (11%) | 504/833 | (61%) | 80/276 | (29%) | 375/572 | (66%) |
|  | I | 103/1087 | (9%) | 108/833 | (13%) | 45/276 | (16%) | 51/572 | (9%) |
|  | II | 433/1087 | (40%) | 169/833 | (20%) | 83/276 | (30%) | 90/572 | (16%) |
|  | III | 289/1087 | (27%) | 38/833 | (5%) | 51/276 | (18%) | 40/572 | (7%) |
|  | IV | 138/1087 | (13%) | 14/833 | (2%) | 17/276 | (6%) | 16/572 | (3%) |
| Dyspnoea status | |  |  |  |  |  |  |  |  |
|  | I | 236/1086 | (22%) | 78/831 | (9%) | 14/276 | (5%) | 125/572 | (22%) |
|  | II | 590/1086 | (54%) | 346/831 | (42%) | 119/276 | (43%) | 251/572 | (44%) |
|  | III | 238/1086 | (22%) | 362/831 | (44%) | 131/276 | (47%) | 178/572 | (31%) |
|  | IV | 22/1086 | (2%) | 45/831 | (5%) | 12/276 | (4%) | 18/572 | (3%) |
| Previous MI | | 538/1063 | (51%) | 31/823 | (4%) | 60/270 | (22%) | 58/563 | (10%) |
| Previous PCI | | 222/1063 | (21%) | 28/819 | (3%) | 39/270 | (14%) | 32/559 | (6%) |
| Previous cardiac surgery | | 9/1116 | (1%) | 112/856 | (13%) | 6/282 | (2%) | 83/601 | (14%) |
| Diabetic | | 322/1080 | (30%) | 107/831 | (13%) | 68/276 | (25%) | 61/568 | (11%) |
|  | Diet controlled | 59/322 | (18%) | 16/107 | (15%) | 16/68 | (24%) | 12/61 | (20%) |
|  | Oral therapy | 163/322 | (51%) | 73/107 | (68%) | 38/68 | (56%) | 37/61 | (61%) |
|  | Insulin controlled | 100/322 | (31%) | 18/107 | (17%) | 14/68 | (21%) | 12/61 | (20%) |
| History of hypertension | | 881/1079 | (82%) | 486/820 | (59%) | 215/270 | (80%) | 306/561 | (55%) |
| Smoking status | |  |  |  |  |  |  |  |  |
|  | Never smoked | 437/1072 | (41%) | 492/827 | (59%) | 114/271 | (42%) | 354/562 | (63%) |
|  | Ex smoker | 523/1072 | (49%) | 289/827 | (35%) | 141/271 | (52%) | 174/562 | (31%) |
|  | Current smoker | 112/1072 | (10%) | 46/827 | (6%) | 16/271 | (6%) | 34/562 | (6%) |
| Preoperative renal failure | | 8/1059 | (1%) | 7/815 | (1%) | 5/273 | (2%) | 4/562 | (1%) |
| History of pulmonary disease | | 121/1074 | (11%) | 88/830 | (11%) | 30/275 | (11%) | 57/565 | (10%) |
| History of neurological disease | | 72/1080 | (7%) | 71/822 | (9%) | 35/275 | (13%) | 49/562 | (9%) |
|  | TIA | 39/69 | (57%) | 43/70 | (61%) | 22/35 | (63%) | 30/49 | (61%) |
|  | CVA (full recovery) | 14/69 | (20%) | 15/70 | (21%) | 10/35 | (29%) | 13/49 | (27%) |
|  | CVA (residual deficit) | 16/69 | (23%) | 12/70 | (17%) | 3/35 | (9%) | 6/49 | (12%) |
| History of neurological dysfunction | | 26/1082 | (2%) | 25/829 | (3%) | 8/273 | (3%) | 14/567 | (2%) |
| Extracardiac arteriopathy | | 121/1065 | (11%) | 67/824 | (8%) | 41/272 | (15%) | 53/560 | (9%) |
| Pre-operative heart rhythm | |  |  |  |  |  |  |  |  |
|  | Sinus rhythm | 1005/1087 | (92%) | 689/835 | (83%) | 222/274 | (81%) | 439/570 | (77%) |
|  | Atrial fibrillation/flutter | 58/1087 | (5%) | 120/835 | (14%) | 46/274 | (17%) | 112/570 | (20%) |
|  | Complete heart block/pacing | 12/1087 | (1%) | 19/835 | (2%) | 4/274 | (1%) | 11/570 | (2%) |
|  | Other abnormal rhythm | 12/1087 | (1%) | 7/835 | (1%) | 2/274 | (1%) | 8/570 | (1%) |
| Left heart catheterisation | | 1167/1177 | (99%) | 751/910 | (83%) | 291/297 | (98%) | 403/633 | (64%) |
| Left main stem disease >50% diameter stenosis* | | 311/1152 | (27%) | 5/797 | (1%) | 32/292 | (11%) | 19/494 | (4%) |
| Ejection fraction | |  |  |  |  |  |  |  |  |
|  | Good (LVEF >50%) | 890/1191 | (75%) | 742/914 | (81%) | 218/302 | (72%) | 509/631 | (81%) |
|  | Fair (LVEF 31 - 50%) | 257/1191 | (22%) | 149/914 | (16%) | 66/302 | (22%) | 105/631 | (17%) |
|  | Poor (LVEF ≤30%) | 44/1191 | (4%) | 23/914 | (3%) | 18/302 | (6%) | 17/631 | (3%) |
| Logistic EuroSCORE *median (IQR)* | | 2.1 | (1.23, 3.59) | 4.0 | (2.08, 6.59) | 5.3 | (3.29, 8.17) | 3.6 | (2.08, 6.23) |

* Other participants had either no left main stem disease or left main stem disease <= 50% diameter

***Missing data*** *[Data given as numbers of participants with missing data overall (numbers with missing data for isolated CABG patients, isolated valve patients, combined CABG and valve patients, other cardiac procedure patients)]:*

**Table A2:** Operative data summarised for each of the operative subgroups in the OMACS cohort. Data are presented as n/N (%) unless otherwise specified.

| Operative details | | Isolated CABG (n=1227) | | Isolated valve (n=946) | | Combined CABG and valve (n=308) | | Other cardiac procedures (n=647) | |
| --- | --- | --- | --- | --- | --- | --- | --- | --- | --- |
|  | |  |  |  |  |  |  |  |  |
| Number of distal coronary anastamoses | |  |  |  |  |  |  |  |  |
|  | 0 | 10/1095 | (1%) |  |  | 7/292 | (2%) | 493/595 | (83%) |
|  | 1 | 94/1095 | (9%) |  |  | 144/292 | (49%) | 44/595 | (7%) |
|  | 2 | 415/1095 | (38%) |  |  | 88/292 | (30%) | 19/595 | (3%) |
|  | 3 | 492/1095 | (45%) |  |  | 49/292 | (17%) | 36/595 | (6%) |
|  | 4+ | 84/1095 | (8%) |  |  | 4/292 | (1%) | 3/595 | (1%) |
| Surgery performed using cardiopulmonary bypass | | 727/1094 | (66%) | 898/900 | (100%) | 292/292 | (100%) | 574/596 | (96%) |
| Cumulative bypass time (minutes) *mean (SD)* | | 75.0 | (59.0, 90.0) | 95.0 | (77.0, 122.0) | 115.0 | (97.5, 138.0) | 129.0 | (95.0, 170.0) |
| Cumulative cross-clamp time (minutes) *median (IQR)* | | 45.0 | (35.0, 54.0) | 70.0 | (56.0, 91.0) | 85.0 | (71.0, 104.0) | 89.0 | (60.0, 119.0) |
| Cold cardioplegia* | | 383/720 | (53%) | 881/885 | (100%) | 290/292 | (99%) | 555/568 | (98%) |
| Antegrade cardioplegia infusion** | | 709/718 | (99%) | 726/883 | (82%) | 201/291 | (69%) | 423/561 | (75%) |
| Intermittent cardioplegia*** | | 717/720 | (100%) | 881/883 | (100%) | 292/292 | (100%) | 562/563 | (100%) |
| Blood cardioplegia**** | | 720/722 | (100%) | 883/887 | (100%) | 292/292 | (100%) | 565/568 | (99%) |
| Tranexamic acid | | 1019/1069 | (95%) | 751/868 | (87%) | 254/272 | (93%) | 407/575 | (71%) |
| Cell saver set up | | 181/1083 | (17%) | 241/881 | (27%) | 75/281 | (27%) | 238/582 | (41%) |
| RBC transfused intra-operatively | | 66/720 | (9%) | 99/881 | (11%) | 53/284 | (19%) | 93/567 | (16%) |
| FFP transfused intra-operatively | | 4/720 | (1%) | 41/881 | (5%) | 28/284 | (10%) | 88/567 | (16%) |
| Platelets transfused intra-operatively | | 112/719 | (16%) | 108/881 | (12%) | 74/285 | (26%) | 182/567 | (32%) |
| Cryoprecipitate transfused intra-operatively | | 12/719 | (2%) | 41/882 | (5%) | 27/284 | (10%) | 123/567 | (22%) |
| Pump blood returned | | 340/691 | (49%) | 407/857 | (47%) | 141/272 | (52%) | 185/546 | (34%) |
| Activation factor VII | | 39/1090 | (4%) | 26/894 | (3%) | 8/287 | (3%) | 10/589 | (2%) |
| Arrhythmias at end of the operation requiring treatment | |  |  |  |  |  |  |  |  |
|  | No, sinus rhythm only | 960/1088 | (88%) | 587/897 | (65%) | 166/291 | (57%) | 369/591 | (62%) |
|  | AV block | 9/1088 | (1%) | 34/897 | (4%) | 16/291 | (5%) | 25/591 | (4%) |
|  | Atrial fibrillation/flutter | 23/1088 | (2%) | 52/897 | (6%) | 19/291 | (7%) | 35/591 | (6%) |
|  | Ventricular fibrillation/ Ventricular tachycardia | 4/1088 | (0.4%) | 9/897 | (1%) | 4/291 | (1%) | 8/591 | (1%) |
|  | Sinus bradycardia | 48/1088 | (4%) | 78/897 | (9%) | 33/291 | (11%) | 48/591 | (8%) |
|  | Other | 44/1088 | (4%) | 137/897 | (15%) | 53/291 | (18%) | 106/591 | (18%) |
| Pacing at end of the operation | |  |  |  |  |  |  |  |  |
|  | None | 973/1086 | (90%) | 557/899 | (62%) | 154/292 | (53%) | 341/590 | (58%) |
|  | Single chamber | 70/1086 | (6%) | 187/899 | (21%) | 74/292 | (25%) | 134/590 | (23%) |
|  | Dual chamber | 31/1086 | (3%) | 125/899 | (14%) | 55/292 | (19%) | 90/590 | (15%) |
|  | Permanent | 11/1086 | (1%) | 26/899 | (3%) | 7/292 | (2%) | 20/590 | (3%) |
|  | Other | 1/1086 | (0%) | 4/899 | (0%) | 2/292 | (1%) | 5/590 | (1%) |
| Total ventilation time (hours) *median (IQR)* | | 10.8 | (9.00, 14.00) | 11.3 | (8.85, 15.50) | 14.0 | (11.10, 21.40) | 12.7 | (9.60, 22.90) |
| Not extubated | | 2/1093 | (0%) | 4/899 | (0%) | 3/292 | (1%) | 7/594 | (1%) |
| Reintubation | | 20/1218 | (2%) | 36/940 | (4%) | 19/304 | (6%) | 49/636 | (8%) |

** All other participants had warm cardioplegia*

*** All other participants had retrograde and antegrade CPB*

**** All other participants had continuous CPB*

***** All other participants had crystalloid or other cardioplegia solution*

***Missing data*** *[Data given as numbers of participants with missing data overall (numbers with missing data for isolated CABG patients, isolated valve patients, combined CABG and valve patients, other cardiac procedure patients)]: Cumulative bypass time - 6 (1, 4, 0, 1); Cumulative cross-clamp time - 21 (6, 10, 0, 5); Total ventilation time - 59 (17, 14, 10, 18)*

*Abbreviations: CABG=coronary artery bypass graft; SD=standard deviation; IQR=interquartile range; RBC=red blood cell; FFP=fresh frozen plasma; AV=Atrioventricular*

**Table A3:** Patient outcomes summarised for each of the operative subgroups in the OMACS cohort. Data are presented as n/N (%) unless otherwise specified.

| Outcomes | Isolated CABG (n=1227) | | Isolated valve (n=946) | | Combined CABG and valve (n=308) | | Other cardiac procedures (n=647) | |
| --- | --- | --- | --- | --- | --- | --- | --- | --- |
| MACE | 18/1218 | (1%) | 25/943 | (3%) | 21/308 | (7%) | 37/643 | (6%) |
| Time to MACE (days) *median (IQR)* | 2.0 | (1.0, 4.0) | 3.0 | (1.0, 5.0) | 6.0 | (1.0, 10.0) | 2.0 | (1.0, 4.0) |
| Confirmed MI | 10/1219 | (1%) | 5/943 | 1%) | 5/307 | (2%) | 6/639 | (1%) |
| Stroke | 7/1219 | (1%) | 11/945 | 1%) | 8/307 | (3%) | 23/640 | (4%) |
| In-hospital death | 4/1226 | (0%) | 9/946 | 1%) | 9/308 | (3%) | 11/647 | (2%) |
| Time to in-hospital death (days) *median (IQR)* | 3.0 | (2.0, 6.5) | 6.0 | (4.0, 46.0) | 10.0 | (2.0, 26.0) | 5.0 | (0.0, 10.0) |
| Death within 1 year | 24/1227 | (2%) | 22/946 | (2%) | 23/308 | (7%) | 21/647 | (3%) |
| Time to death (within 1 year) (days) *median (IQR)* | 212.0 | (33.0, 295.5) | 46.5 | (8.0, 137.0) | 71.0 | (13.0, 138.0) | 14.0 | (5.0, 176.0) |
| Time to ICU discharge (hours) *median (IQR)* | 66.2 | (43.9, 91.3) | 68.9 | (49.9, 111.5) | 91.0 | (65.6, 141.3) | 88.5 | (62.5, 135.7) |
| Time to hospital discharge (days) *median (IQR)* | 6.0 | (5.0, 8.0) | 7.0 | (6.0, 10.0) | 8.0 | (6.0, 12.0) | 8.0 | (6.0, 14.0) |
| Reoperation | 38/1220 | (3%) | 54/945 | (6%) | 18/307 | (6%) | 48/642 | (7%) |
| Reoperation for bleeding | 28/32 | (88%) | 28/44 | (64%) | 9/15 | (60%) | 23/35 | (66%) |
| Reoperation for other reasons | 8/32 | (6%) | 29/44 | (27%) | 11/15 | (7%) | 29/35 | (23%) |
| Cardiac arrest | 8/1219 | (1%) | 11/945 | (1%) | 6/307 | (2%) | 11/640 | (2%) |
| Cardiac arrest resuscitation attempted | 6/8 | (75%) | 11/11 | (100%) | 5/6 | (83%) | 10/11 | (91%) |
| Cardiac arrest resuscitation successful | 5/6 | (83%) | 9/11 | (82%) | 5/5 | (100%) | 7/10 | (70%) |
| SVT/AF | 365/1219 | (30%) | 350/945 | (37%) | 134/307 | (44%) | 229/640 | (36%) |
| VT/VF | 8/1219 | (1%) | 13/945 | (1%) | 4/307 | (1%) | 11/640 | (2%) |
| New pacing | 126/1219 | (10%) | 348/945 | (37%) | 130/307 | (42%) | 255/640 | (40%) |
| Temporary pacing became permanent | 13/119 | (11%) | 44/334 | (13%) | 11/126 | (9%) | 43/236 | (18%) |
| ICD | 11/827 | (1%) | 5/713 | (1%) | 1/216 | (0%) | 7/489 | (1%) |
| Vasopressors used | 686/1094 | (63%) | 544/899 | (61%) | 215/291 | (74%) | 391/590 | (66%) |
| Any inotropes used | 229/1094 | (21%) | 330/899 | (37%) | 123/291 | (42%) | 242/590 | (41%) |
| IABP inserted | 14/1219 | (1%) | 4/945 | (0%) | 7/307 | (2%) | 8/640 | (1%) |
| Pulmonary artery catheter inserted | 6/1219 | (0%) | 10/945 | (1%) | 9/307 | (3%) | 9/640 | (1%) |
| Vasodilator used | 188/1219 | (15%) | 267/945 | (28%) | 64/307 | (21%) | 159/640 | (25%) |
| Tracheostomy | 6/1219 | (0%) | 11/945 | (1%) | 7/307 | (2%) | 12/640 | (2%) |
| Mask CPAP | 93/1173 | (8%) | 67/899 | (7%) | 38/291 | (13%) | 66/601 | (11%) |
| ARDS | 3/1219 | (0%) | 3/945 | (0%) | 1/307 | (0%) | 5/640 | (1%) |
| Pneumothorax or pleural effusion requiring drainage | 27/1094 | (2%) | 34/899 | (4%) | 17/291 | (6%) | 37/589 | (6%) |
| High flow oxygen | 94/638 | (15%) | 85/621 | (14%) | 41/156 | (26%) | 89/420 | (21%) |
| Haemofiltration/dialysis since heart operation | 16/1174 | (1%) | 16/899 | (2%) | 11/291 | (4%) | 27/601 | (4%) |
| Acute kidney injury | 256/1058 | (24%) | 194/884 | (22%) | 99/287 | (34%) | 163/581 | (28%) |
| Stage 1 | 195/256 | (76%) | 127/194 | (65%) | 73/99 | (74%) | 106/163 | (65%) |
| Stage 2 | 36/256 | (14%) | 40/194 | (21%) | 14/99 | (14%) | 21/163 | (13%) |
| Stage 3 | 25/256 | (10%) | 27/194 | (14%) | 12/99 | (12%) | 36/163 | (22%) |
| Peptic ulcer/GI bleed/perforation | 1/1219 | (0%) | 5/945 | (1%) | 2/307 | (1%) | 4/640 | (1%) |
| Pancreatitis | 0/1219 | (0%) | 1/945 | (0%) | 0/307 | (0%) | 1/640 | (0%) |
| Ischaemic bowel requiring treatment | 1/1219 | (0%) | 0/945 | (0%) | 0/307 | (0%) | 1/640 | (0%) |
| TIA | 0/1219 | (0%) | 4/945 | (0%) | 3/307 | (1%) | 3/640 | (0%) |
| DVT | 2/1219 | (0%) | 1/945 | (0%) | 2/307 | (1%) | 3/640 | (0%) |
| Pulmonary embolus | 1/1219 | (0%) | 1/945 | (0%) | 1/307 | (0%) | 6/640 | (1%) |
| Excess bleeding not requiring re-operation | 18/1094 | (2%) | 12/899 | (1%) | 8/291 | (3%) | 8/590 | (1%) |
| Pericardial effusion requiring drainage | 22/1174 | (2%) | 50/899 | (6%) | 19/290 | (7%) | 41/601 | (7%) |
| Any unexpected complication | 203/1227 | (17% | 193/946 | (20%) | 88/307 | (29%) | 161/644 | (25%) |
| ***Participants with direct data entry in OMACS*** |  |  |  |  |  |  |  |  |
| *Any suspected infection* | *324/963* | *(34%)* | *295/774* | *(38%)* | *124/257* | *(48%)* | *254/534* | *(48%)* |
| *Any confirmed infection (OMACS data entry only)* | *53/818* | *(6%)* | *64/682* | *(9%)* | *36/206* | *(17%)* | *53/469* | *(11%)* |
| *Suspected sepsis* | *221/1094* | *(20%)* | *230/899* | *(26%)* | *84/291* | *(29%)* | *213/590* | *(36%)* |
| *Temperature <36C or >38C* | *185/221* | *(84%)* | *189/230* | *(82%)* | *70/84* | *(83%)* | *177/213* | *(83%)* |
| *Unexplained increased heart rate above normal for patient* | *57/221* | *(26%)* | *51/229* | *(22%)* | *18/83* | *(22%)* | *53/213* | *(25%)* |
| *CRP >5mg/L* | *218/221* | *(99%)* | *227/230* | *(99%)* | *84/84* | *(100%)* | *210/213* | *(99%)* |
| *WBC >12.0* | *105/221* | *(48%)* | *91/230* | *(40%)* | *49/84* | *(58%)* | *100/213* | *(47%)* |
| *Unexplained increased respiratory rate above normal for patient* | *79/220* | *(36%)* | *76/229* | *(33%)* | *29/83* | *(35%)* | *67/213* | *(31%)* |
| *Respiratory infection* | *201/960* | *(21%)* | *167/767* | *(22%)* | *93/256* | *(36%)* | *144/529* | *(27%)* |
| *Superficial wound infection* | *39/952* | *(4%)* | *23/760* | *(3%)* | *28/255* | *(11%)* | *30/527* | *(6%)* |
| *Mediastinitis* | *0/953* | *(0%)* | *0/760* | *(0%)* | *1/255* | *(0%)* | *1/527* | *(0%)* |
| *Wound dehiscence requiring rewiring or treatment* | *6/952* | *(1%)* | *9/761* | *(1%)* | *9/255* | *(4%)* | *3/527* | *(1%)* |
| *UTI* | *16/953* | *(2%)* | *13/761* | *(2%)* | *4/255* | *(2%)* | *9/527* | *(2%)* |
| *Unspecified infection* | *10/952* | *(1%)* | *13/761* | *(2%)* | *5/254* | *(2%)* | *18/526* | *(3%)* |
| *Other infection* | *11/633* | *(2%)* | *21/565* | *(4%)* | *6/153* | *(4%)* | *18/398* | *(5%)* |
| *Post-op antibiotics started* | *231/322* | *(72%)* | *191/295* | *(65%)* | *99/124* | *(80%)* | *193/254* | *(76%)* |

***Missing data*** *[Data given as numbers of participants with missing data overall (numbers with missing data for isolated CABG patients, isolated valve patients, combined CABG and valve patients, other cardiac procedure patients)]: Time to ICU discharge - 51 (17, 11, 9, 14);*

*Abbreviations: MACE=major adverse cardiovascular event; IQR=inter-quartile range; MI=myocardial infarction; ICU=intensive care unit; SVT/AF=supraventricular tachycardia/atrial fibrillation; VT/VF=ventricular tachycardia/ventricular fibrillation; ICD=* *implantable cardioverter defibrillator; IABP=intra-aortic balloon pump; CPAP=continuous positive airway pressure; ARDS=acute respiratory distress syndrome; GI=gastrointestinal; TIA=transient ischaemic attack; DVT=deep vein thrombosis; CRP=c-reactive protein; WBC=white blood cell; UTI=urinary tract infection.*

**Table A4:** Patient quality of life outcomes summarised for each of the operative subgroups in the OMACS cohort. Data are presented as n/N (%) unless otherwise specified.

| Outcomes | Isolated CABG (n=1469) | | Isolated valve (n=1127) | | Combined CABG and valve (n=352) | | Other cardiac procedures (n=735) | |
| --- | --- | --- | --- | --- | --- | --- | --- | --- |
| SF12 questionnaires at 3 months |  |  |  |  |  |  |  |  |
| Questionnaires returned | 5/6 | 83% | 494/653 | 76% | 17/19 | 89% | 273/372 | 73% |
| Physical component score *median (IQR)* | 52.3 | (51.94, 56.05) | 47.0 | (39.97, 53.82) | 42.2 | (38.87, 51.52) | 46.0 | (38.67, 52.88) |
| Mental component score *median (IQR)* | 50.0 | (49.47, 50.08) | 51.7 | (43.36, 58.33) | 47.7 | (39.56, 57.55) | 51.0 | (41.15, 57.21) |
| SF12 questionnaires at 12 months |  |  |  |  |  |  |  |  |
| Questionnaires returned | 7/7 | 100% | 446/674 | 66% | 9/17 | 53% | 243/398 | 61% |
| Physical component score *median (IQR)* | 55.7 | (54.42, 58.38) | 49.5 | (40.58, 56.13) | 46.9 | (39.46, 52.75) | 50.6 | (40.50, 55.63) |
| Mental component score *median (IQR)* | 55.3 | (54.59, 57.53) | 53.2 | (43.36, 58.59) | 54.7 | (47.31, 58.09) | 52.4 | (42.75, 57.53) |
| Coronary Revascularisation Outcomes Questionnaire at 3 months |  |  |  |  |  |  |  |  |
| Questionnaires returned | 734/920 | 80% | 5/8 | 62% | 137/176 | 78% | 70/96 | 73% |
| Symptoms score *median (IQR)* | 92.9 | (85.71, 100.00) | 89.3 | (78.57, 96.43) | 92.9 | (85.71, 100.00) | 92.9 | (82.73, 96.43) |
| Physical score *median (IQR)* | 93.8 | (75.00, 100.00) | 71.9 | (40.62, 100.00) | 87.5 | (68.75, 93.75) | 87.5 | (75.00, 100.00) |
| Cognitive function score *median (IQR)* | 93.3 | (80.00, 100.00) | 80.0 | (80.00, 100.00) | 93.3 | (80.00, 100.00) | 93.3 | (80.00, 100.00) |
| Psychological function score *median (IQR)* | 85.7 | (69.64, 94.64) | 75.0 | (71.43, 85.71) | 83.9 | (70.83, 94.64) | 82.1 | (69.23, 92.86) |
| Satisfaction score *median (IQR)* | 83.3 | (69.44, 93.33) | 77.8 | (72.22, 87.50) | 83.3 | (67.36, 91.67) | 77.2 | (67.36, 83.33) |
| Adverse events score *median (IQR)* | 88.6 | (77.27, 95.45) | 93.2 | (84.09, 95.45) | 88.6 | (81.82, 95.45) | 88.6 | (75.00, 97.73) |
| Coronary Revascularisation Outcomes Questionnaire at 12 months |  |  |  |  |  |  |  |  |
| Questionnaires returned | 702/946 | 74% | 14/18 | 78% | 146/200 | 73% | 71/95 | 75% |
| Symptoms score *median (IQR)* | 96.4 | (85.71, 100.00) | 92.3 | (86.29, 96.43) | 96.4 | (85.71, 100.00) | 92.9 | (85.71, 100.00) |
| Physical score *median (IQR)* | 100.0 | (81.25, 100.00) | 90.6 | (25.00, 100.00) | 93.8 | (75.00, 100.00) | 93.8 | (75.00, 100.00) |
| Cognitive function score *median (IQR)* | 93.3 | (80.00, 100.00) | 86.7 | (80.00, 100.00) | 93.3 | (86.67, 100.00) | 93.3 | (73.33, 100.00) |
| Psychological function score *median (IQR)* | 91.1 | (78.57, 96.43) | 85.7 | (75.00, 92.86) | 94.6 | (80.36, 98.21) | 91.1 | (76.92, 96.43) |
| Satisfaction score *median (IQR)* | 83.3 | (70.00, 95.83) | 70.4 | (50.00, 83.33) | 83.3 | (61.11, 91.67) | 83.3 | (65.28, 91.67) |
| Adverse events score *median (IQR)* | 93.2 | (86.36, 97.73) | 95.5 | (86.36, 97.73) | 95.5 | (90.91, 100.00) | 95.5 | (86.36, 100.00) |

***Missing data*** *[Data given as numbers of participants with missing data overall (number of participants with samples with missing data)]: SF12 3 month PCS - 7 (0, 5, 0, 2); SF12 3 month MCS - 7 (0, 5, 0, 2); SF12 12 month PCS - 6 (0, 4, 0, 2); SF12 12 month MCS - 6 (0, 4, 0, 2); CROQ 3 month symptoms score - 3 (1, 0, 1, 1); CROQ 3 month physical score - 12 (7, 1, 4, 0); CROQ 3 month cognitive function score - 7 (4, 0, 2, 1); CROQ 3 month psychological function score - 7 (5, 0, 2, 0); CROQ 3 month satisfaction score - 3 (1, 0, 1, 1); CROQ 3 month adverse events score - 13 (9, 0, 1, 1); CROQ 12 month symptoms score - 4 (2, 0, 0, 2); CROQ 12 month physical score - 13 (11, 0, 1, 1); CROQ 12 month cognitive function score - 6 (4, 0, 1, 1); CROQ 12 month psychological function score - 7 (5, 0, 0, 2); CROQ 12 month satisfaction score - 6 (4, 0, 1, 1); CROQ 12 month adverse events score - 13 (8, 0, 4, 1)*

*Abbreviations: CABG=Coronary artery bypass grafting;* *IQR=interquartile range*
