# Supplementary material for: Cohort profile: Outcome Monitoring After Cardiac Surgery (OMACS) – a prospective UK cohort study of cardiac surgery patients at the Bristol Heart Institute
Source: BMJ Open. 2025 Feb 5;15(2):e091518. doi: 10.1136/bmjopen-2024-091518 (PMC11800208; doi:10.1136/bmjopen-2024-091518)
Supplement: Supplementary file 2 [file bmjopen-2024-091518.reviewer_comments.pdf]

PEER REVIEW HISTORY

BMJ Open publishes all reviews undertaken for accepted manuscripts. Reviewers are asked to complete a checklist review form and are provided with free text boxes to elaborate on their assessment. These free text comments are reproduced below.

ARTICLE DETAILS

Title (Provisional)

Cohort profile: Outcome Monitoring after Cardiac Surgery (OMACS): a prospective UK cohort study of cardiac surgery patients at the Bristol Heart Institute

Authors

Walker-Smith, Terrie; Hopkins, Emma; Lambert, Penny; Joyce, Katherine; Maishman, Rachel; Smartt, Helena; Angelini, Gianni; Rogers, Chris A; Reeves, Barnaby; Brierley, Rachel; Culliford, Lucy

---

VERSION 1 - REVIEW

**Reviewer**	**1**
**Name**	**Jeppsson, Anders**
**Affiliation**	**University of Gothenburg, Cardiothoracic Surgery**
**Date**	**17-Oct-2024**
**COI**	**None**

This manuscript describes the build-up of a large database and biobank (OMAC) with over 4000 cardiac surgery patients. The database includes patient characteristics, operative details, quality of life (QoL) questionnaires, and information on mortality. The data and samples will be available for external researchers on reasonable request. The authors should be congratulated and commended for their effort, which is really extraordinary.

1. The subject is interesting. However, the manuscript is overloaded with details about e.g. type of test tube, patient characteristics and operative details, both in text and in tables. Furthermore, the authors have included a description and results of two studies on patient information and how these impacts study recruitment. Taken together, these parts make the manuscript difficult to read. I suggest that the manuscript focus on the rationale and build-up of the database and the biobank, the comparison with other databases, and how the data can be used in the future. Details on patient data and surgery can be better reported in a supplement or in a separate manuscript.

2. The manuscript also report e.g. changes in patient characteristics and surgical approach over time, and the results of the QoL questionnaires. Although interesting, this needs to be reported in separate paper(s) where more in-depth analyses of the results can be applied.

3. Please clarify earlier in the manuscript the application process and demands for external researches who want to get access to data.

4. There are numerous abbreviations used in the text. Pleas provide a list of abbreviation in the beginning of the manuscript.

5. Are long-term outcome variables, such as mortality, reinterventions, post-discharge myocardial infarction and stroke, available for the patients? Does the database contain information about socioeconomic variables and postoperative medication? Please clarify and consider to add a short limitation paragraph.

Again, the authors should be applauded for their effort.

**Reviewer**	**2**
**Name**	**McCluskey, Stuart**
**Affiliation**	**University Health Network, Anesthesia and Pain Management**
**Date**	**18-Oct-2024**
**COI**	**None**

The authors should be congratulated for their efforts to collect outcome data and biological samples from an important patient population. The value of the available date clearly has potential, but I do not understand the need to describe the data the authors have collected. A rich set of data and the offer to make this information available to others is interesting. However, what is described in the manuscript would not move our group any closer to requested data from the authors.

I would like to know how many patients were approached and how many signed consent and I would be interested to know whey the data collections stopped.

Minor Point. Our research ethic committee does not allow people in the circle of care to also recruit or consent patients. I do not believe we could use the data.

VERSION 1 - AUTHOR RESPONSE

Reviewer: 1
Dr. Anders Jeppsson, University of Gothenburg
Comments to the Author:
This manuscript describes the build-up of a large database and biobank (OMAC) with over 4000 cardiac surgery patients. The database includes patient characteristics, operative details, quality of life (QoL) questionnaires, and information on mortality. The data and samples will be available for external

researchers on reasonable request. The authors should be congratulated and commended for their effort, which is really extraordinary.

1. The subject is interesting. However, the manuscript is overloaded with details about e.g. type of test tube, patient characteristics and operative details, both in text and in tables. Furthermore, the authors have included a description and results of two studies on patient information and how these impacts study recruitment. Taken together, these parts make the manuscript difficult to read. I suggest that the manuscript focus on the rationale and build-up of the database and the biobank, the comparison with other databases, and how the data can be used in the future. Details on patient data and surgery can be better reported in a supplement or in a separate manuscript.
Response: We have not made this change. The reasons are that we believe that details such as the way in which bloods were taken and processed and the patient characteristics and operative details are important details for anyone who may wish to request use the samples. These details indicate if the sample will be suitable for the analysis they have planned and if we have samples for the types of patients or operations that the researcher is interested in (e.g. a researcher may want to compare samples form those who had a mitral valve operation who did / did not suffer myocardial infarction). The results of the 2 studies within a trial were included as the instructions for authors requests a brief description of any results that already known.

2. The manuscript also report e.g. changes in patient characteristics and surgical approach over time, and the results of the QoL questionnaires. Although interesting, this needs to be reported in separate paper(s) where more in-depth analyses of the results can be applied.
Response: Many apologies but I'm not sure which parts of the manuscript the first part of this comment relates to? We haven't described any changes in our cohort over time. We have included some summary measures of the QoL data, but similar to the response to point 1, this is simply to describe the characteristics of the cohort. No analyses is planned as this would be the work of future research projects using these data.

3. Please clarify earlier in the manuscript the application process and demands for external researches who want to get access to data.
Response: We have added to the abstract and the main text as note to say that requests should be made to the corresponding author. We are not able to expand on the requirements beyond to say that 'Appropriate research ethics committee approval must be in place for use of the OMACS data and samples by secondary researchers.' as this is likely to be different by institution and country (for example researchers within my own institution, the University of Bristol, would apply to our faculty research ethics committee.

4. There are numerous abbreviations used in the text. Pleas provide a list of abbreviation in the beginning of the manuscript.
Response: We have defined the abbreviations in the text the first time that they are used as per the guidance for authors. A list of abbreviations is not part of the article template and so has not been included. We can add this if the editors agree.

5. Are long-term outcome variables, such as mortality, reinterventions, post-discharge myocardial infarction and stroke, available for the patients? Does the database contain information about socioeconomic variables and postoperative medication? Please clarify and consider to add a short limitation paragraph.
Response: these data are not available, all data that are available has been listed in the tables. We have added a sentence to say that long-term post-discharge outcomes are not available and another to say that further data linkage is not possible restricting the data items to those listed.

Again, the authors should be applauded for their effort.
Response: Thank you

Reviewer: 2
Dr. Stuart McCluskey, University Health Network, University of Toronto
Comments to the Author:
The authors should be congratulated for their efforts to collect outcome data and biological samples from

an important patient population. The value of the available date clearly has potential, but I do not understand the need to describe the data the authors have collected. A rich set of data and the offer to make this information available to others is interesting. However, what is described in the manuscript would not move our group any closer to requested data from the authors.

I would like to know how many patients were approached and how many signed consent and I would be interested to know whey the data collections stopped.

Response: Thank you, we know from the requests that we've already had to use these data and samples that knowledge of the patient characteristics and operative details can be important to know if relevant data and samples are included. The guidance for authors requests that the cohort are described. The numbers approached and consented are described on page 6, and we have added a sentence to explain why data collection stopped.

Minor Point. Our research ethic committee does not allow people in the circle of care to also recruit or consent patients. I do not believe we could use the data.
Response: thank you for this point which may prevent the use of these data and samples by some researchers. In the UK it is a requirement for the recruitment and consent of research participants to be made by someone form the direct care team as personal data cannot be revealed to those outside the care team without consent in place.

VERSION 2 - REVIEW

**Reviewer**	**1**
**Name**	**Jeppsson, Anders**
**Affiliation**	**University of Gothenburg, Cardiothoracic Surgery**
**Date**	**22-Dec-2024**
**COI**	

Please see my previous review. I still think that the manuscript can be enhanced by focusing on the database in itself rather than patient data.
